# Vegetation structure and aboveground biomass of Páramo peatlands along a high-elevation gradient in the northern Ecuadorian Andes

**DOI:** 10.3389/fpls.2023.1102340

**Published:** 2023-05-08

**Authors:** Esteban Suárez, John A. Hribljan, Segundo Chimbolema, Katie Harvey, Victoria Triana, Juan E. Zurita, Ricardo Jaramillo, Lenka G. Doskocil

**Affiliations:** ^1^ Colegio de Ciencias Biológicas y Ambientales, Universidad San Francisco de Quito, Quito, Ecuador; ^2^ Instituto Biósfera, Universidad San Francisco de Quito, Quito, Ecuador; ^3^ Department of Biology, University of Nebraska Omaha, Omaha, NE, United States

**Keywords:** plant communities, aboveground biomass, Ecuador, Páramo, peatlands, elevation gradient

## Abstract

The high-elevation peatlands of the páramos of the northern Andes constitute a diverse environment that harbors large numbers of species and several types of plant communities along altitudinal, latitudinal, and environmental gradients. However, little is known about the structure and functioning of these ecosystems, including peatland vegetation types and their relative contribution to the production and accumulation of peat soils. In this paper we characterized the structure of peatland plant communities of the humid páramos of northern Ecuador by describing the distribution of plant growth-forms and their aboveground biomass patterns. Along an elevation gradient of 640 m we sampled vegetation in 16 peatlands and aboveground biomass in four peatlands. Three distinct peatland vegetation types were identified: High elevation Cushion peatlands, dominated by *Plantago rigida* and *Distichia muscoides*, Sedge and rush peatlands dominated by *Carex* spp. and *Juncus* spp., and Herbaceous and shrubby peatlands, with a more heterogenous and structurally complex vegetation. In terms of aboveground biomass, we found an 8-fold reduction in the higher peatlands compared to the lower sites, suggesting that the steep elevational gradients characteristic of Andean environments might be crucial in structuring the physiognomy and composition of peatland vegetation, either through its effects on temperature and other environmental factors, or through its effects on the age and development of soils. Additional studies are needed to evaluate the potential effects of temperature, hydrology, micro-topography, geological setting, and land-use, which are likely to influence vegetation patters in these peatlands.

## Introduction

The South American páramos are a striking biome of the northern Andes. Guarding the highest mountain peaks of Venezuela, Colombia, Ecuador and northern Perú, these complex and diverse ecosystems emerge above the tree-line covering vast areas shaped by a long history of glacial and, in some regions, volcanic activity (Balslev and Luteyn, 1992). The resulting topography is tortuous, crisscrossed by deep ravines, glacial terraces, and steep slopes where the strong elevation gradient (roughly between 3200 m and the lower limit of glaciers) drastically affects the nature of biotic communities (Hofstede et al., 2014). Páramo ecosystems have been widely recognized for their extremely high levels of biodiversity and endemism, but also as natural laboratories to understand biogeographical and evolutionary patterns that control the development and distribution of high-elevation plant species and their sensitivity to climate change (Madriñán et al., 2013; Anthelme et al., 2014; Sanchez-Baracaldo and Thomas, 2014). Specifically, the physiognomy and species composition of plant communities and the role of soils in regulating hydrological flows have been extensively studied with emphasis on the impacts of burning, grazing, expanding agriculture, afforestation and, more recently, climate change (Hofstede and Rossenaar, 1995; Suárez and Medina, 2001; Farley et al., 2004; Anthelme et al., 2021; Lasso et al., 2021; Thompson et al., 2021). However, most studies have only considered well-drained upland soils, thus overlooking a vast and complex system of páramo peatlands that, in some areas, can cover up to 25% of the páramo landscape (Hribljan et al., 2017) and potentially contribute substantially to the diversity and functioning of páramo ecosystem. Previous research on these peatlands of the northern Andes has been mostly focused on the patterns of carbon dynamics and storage (Cooper et al., 2015; Hribljan et al., 2016; Hribljan et al., 2017), the environmental factors that control the distribution of peat forming species, and the high rates of peat accumulation reported for these environments (Benavides et al., 2013; Benfield et al., 2021). However, little is known about the structure and functioning of these ecosystems, including critical information regarding the distribution of different peatland vegetation types and their relative contribution to the production and accumulation of peat soils. In this paper we offer a first attempt at understanding the composition, structure, and aboveground biomass patterns of high-elevation peatland communities along an elevation gradient in the páramos of northern Ecuador.

Research on the vegetation of páramo peatlands is limited to a few studies that emphasized the description of vegetation types, and patterns of carbon dynamics. These studies have reported large heterogeneity in plant community structure, mostly related to elevation, the chemical characteristics of ground water inputs into the peatlands and other environmental (*e.g*. water level) and anthropogenic disturbance gradients (Schmidt-Mumm and Vargas Ríos, 2012; Ruthsatz et al., 2020; Benavides et al., 2023). Deil et al. (2011) defined eleven plant associations for peatlands along the Andes, including two associations (*Muhlenbergia fastigiata*-*Distichlis* and *Gentiana sedifolia*-*Carex*) that coarsely coincide with some peatland plant communities of the northern Andes. In contrast, Schmidt-Mumm and Vargas Ríos (2012) recognized eighteen separate plant communities that occur across terrestrial-aquatic transition in the páramo of Chingaza in central Colombia.

Páramo peatlands have also been recognized for their high rates of peat-accumulation, which are among the highest for mountain peatlands worldwide (Chimner and Karberg, 2008; Benavides et al., 2013; Hribljan et al., 2016). The rapid peat-growth rates in these peatlands are attributed to highly productive vegetation communities and, in recent geological times, to increased mineral nutrients and warmer temperatures (Hribljan et al., 2016; Benfield et al., 2021; Suárez et al., 2021). Therefore, plant community structure and growth are significant factors in the functioning of these peatlands both as a source of organic matter and as a protective layer for the underlying peat soils.

In a previous study on north-eastern Ecuador’s páramo peatlands we offered a first evaluation of peatland types found at elevations above 3500 m (Hribljan et al., 2017). By using field surveys and a multi-date, multi-sensor technique, we were able to delineate three main types of páramo peatlands and discern their individual contribution to soil carbon storage at the landscape level. Despite the accuracy of the mapping technique that we used, questions remain about the precision of that classification at smaller scales, and about the structure and composition of those vegetation types. Moreover, additional information is needed to explore the environmental factors that might control the different peatland vegetation types found in this part of the northern Andes. This information is essential to future studies focusing on water regulation mechanisms and carbon accumulation patterns in these ecosystems, as well as conservation and restoration of an ecosystem that has been severely damaged by grazing, drainage, and land-use change across its distribution (Suárez et al., 2022a). From this perspective, the main objectives of this study are to i) contribute to the description of the composition and structure of peatland plant communities of the northern Ecuador’s humid páramos and ii) describe their aboveground biomass patterns. For this exploratory study we described the structure of the plant communities by characterizing the coverage of the main plant growth forms (*sensu* Ramsay and Oxley, 1997) because they might be more useful than individual species in terms of exploring the physiognomy and functional attributes of the peatlands (*e.g*. carbon storage). Ultimately our goal is to provide a basis for better management and future research in a critical component of the páramo ecosystems that has been largely overlooked and plays a significant role in the ecosystem services provided by this environment.

## Materials and methods

### Study area

This study was conducted in the Chakana caldera, a volcanic system spanning roughly 50 km in the Eastern branch of Ecuador’s northern Andes with the last volcanic activity registered in the 1700s (Hall and Mothes, 2008). This area is characterized by carbon dense organic soils interbedded with volcanic ash and pumice layers (Hribljan et al., 2016). Glacial activity across the greater landscape created a complex and steep topography that contributes additional mineral inputs from side slope erosion. Our study sites straddle the Andean continental divide, at elevations between 3700 and 4340 m, and experience mean annual temperatures that range from 6°C at 3600 m, to 3.7°C at 4300 m (Suarez E., unpublished data). Precipitation at 3600 m can reach 1300 to 1700 mm yr^-1^, but clouds and mist coming from the Amazon basin supply a large amount of water that still needs to be quantified. The steep slopes of this volcanic complex are typically dominated by tussock grasses (*e.g*. *Calamagrostis* spp., *Festuca* spp.) interspersed with shrubs (*e.g*. *Monticalia*, *Hypericum*, *Diplostephium* spp., *Valeriana* spp.), and a rich layer of herbs and scandent vegetation including characteristic species of the genera *Hypochaeris*, *Werneria*, *Senecio*, and *Phlegmariurus*, among others. In the midst of these grasslands, glacial and volcanic activity carved a complex system of peatlands that, in this particular area, cover at least 24% of the landscape between 3500 and 4300 m (Hribljan et al., 2017).

For this study we analyzed vegetation patterns at 16 individual peatlands spanning diverse geographical settings and located between 3700 and 4340 m in the Cayambe-Coca National Park and its buffer zone (0° 19.417’S, 78° 12.180’W; 0° 14.370’S, 78° 7.929’W). In order to ensure a representative sample of the peatlands in this region, we selected sites that i) represented the elevation gradient of the study area, ii) showed little or no signs of recent human disturbance, and iii) encompassed all the general vegetation types that had been reported in the region (Hribljan et al., 2017). However, it must be noted that our selection of sites was largely biased towards peatlands that occurred within 5 km of roads. Peatlands which occurred alongside of a road and showed signs of potential hydrological impacts were not included. Additionally, four of those sites, exhibiting similar slope, aspect, and general topography within the same watershed and showing no signs of recent human disturbance, were selected to characterize altitudinal changes in aboveground biomass.

### Plant community characterization

Percent cover and taxonomic identity of vascular and non-vascular plant species present in a set of eight randomly located plots (1x1 m) within each peatland were recorded. Vegetation data from plots were summarized by growth form (Ramsay and Oxley, 1997) and classified using hierarchical, polythetic, agglomerative cluster analysis using Sørensen distance measure and flexible beta linkages method with β= -0.25 using *Vegan* package in R 4.1.1 (Oksanen et al., 2022). None of the growth form categories proposed by Ramsay and Oxley (1997) were adequate to describe the stands of graminoid species which do not form tussocks and occur in more or less continuous stands or matts in poorly drained areas. To account for these species, we divided the tussock growth form into two groups: tussock graminoids (*Calamagrostis* spp., *Festuca* spp., and *Cortaderia* spp.) and non-tussock graminoids (*e.g. Bromus lanatus*, *Agrostis* spp., *Carex* spp., *Eleocharis* spp., *Uncinia* spp., and *Juncus* spp.). Indicator species analysis coupled with Monte Carlo Analysis provided a quantitative, objective criterion to prune the dendrogram and optimize the number of clusters (McCune et al., 2002; De Cáceres and Legendre, 2009). The cluster level with the smallest average p-value derived from the indicator species analysis was used as the optimal clustering level. Dominant growth forms for each group were determined using the Indicator Value Index (IndVal) component B while indicative growth forms were determined using IndVal component A (De Cáceres and Legendre, 2009).

In order to support the results of the cluster analysis, ordination of vegetation communities was also conducted on non-transformed data using non-metric Multidimensional Scaling (nMDS). The ordination solution was obtained in R^®^ 4.1.1 using Bray–Curtis distance measure and the r-function metaMDS for constructing multiple runs, finding the best solution for each dimensionality with a stress less than 0.2.

### Aboveground biomass

Plant biomass varies widely across species and environmental conditions, which means that gathering field-based ecosystem-scale estimates of aboveground biomass can be challenging. From this perspective, for this study we used plant growth forms (*sensu* Ramsay and Oxley, 1997) to estimate aboveground biomass. Plant growth forms are an attempt to group species that share architectural and functional characteristics which probably emerged among different species as a comparable response to significant environmental constraints. Our use of plant growth forms is based on the assumption that the variation in aboveground biomass will be lower within growth-form categories, than among individual species, or taxonomic groups.

Altitudinal changes in aboveground biomass were examined in four of the 16 peatlands located at 3920, 4125, 4240, and 4300 m. An additional measurement of plant cover in these four peatlands was conducted using a line intercept method (Floyd and Anderson, 1987; Carlsson et al., 2005; Rochefort et al., 2013) along 10 m long transects that were positioned in a stratified arrangement. This additional measurement of plant cover was intended to provide a more detailed measurement of the variability in plant community structure present at these sites. The number of transects measured in each site varied between 10 and 22, depending on the size of the peatland and the heterogeneity of plant cover. Along each transect we recorded the distance of the transect that intersected different growth forms and the dominant species of each one.

Aboveground biomass in these four peatlands was estimated by combining percent growth-form cover determined from the transect surveys with plant biomass estimates for each representative growth form found at each elevation. Polygons for each peatland were generated using a Garmin 60 CX GPS and uploaded into ArcGis^®^ where we estimated peatland surface area using the assumption that all our sites were essentially flat. The average percent cover along the transects was used to calculate the proportion of each peatland’s area occupied by the eleven different plant growth forms. We then collected four to six samples of each growth form present in each site using a 30 x 30 cm PVC quadrat randomly positioned over representative individuals or patches of the most frequent species of each growth form, based on the data collected along the transects described above. For each sample, we clipped all aerial biomass within the quadrat down to where the first roots appeared on the stems. In the case of shrubs and tussock grasses, we excluded any aerial biomass that projected outside of the frame of the quadrat. Plant biomass samples were placed in bags, transported to the Laboratory of Aquatic Ecology at Universidad San Francisco de Quito, and dried at 65°C to a constant weight. The samples were then cut and homogenized, and replicate subsamples were reduced to ash in a muffle furnace (550°C for 5 hours). The ash content was used to calculate ash-free biomass of each sample. Finally, mean aboveground biomass of each peatland was calculated using the average biomass contributed by each growth form according to their proportional percentage of ground cover in each site.

## Results

### Vegetation community structure

**Figure 1:**
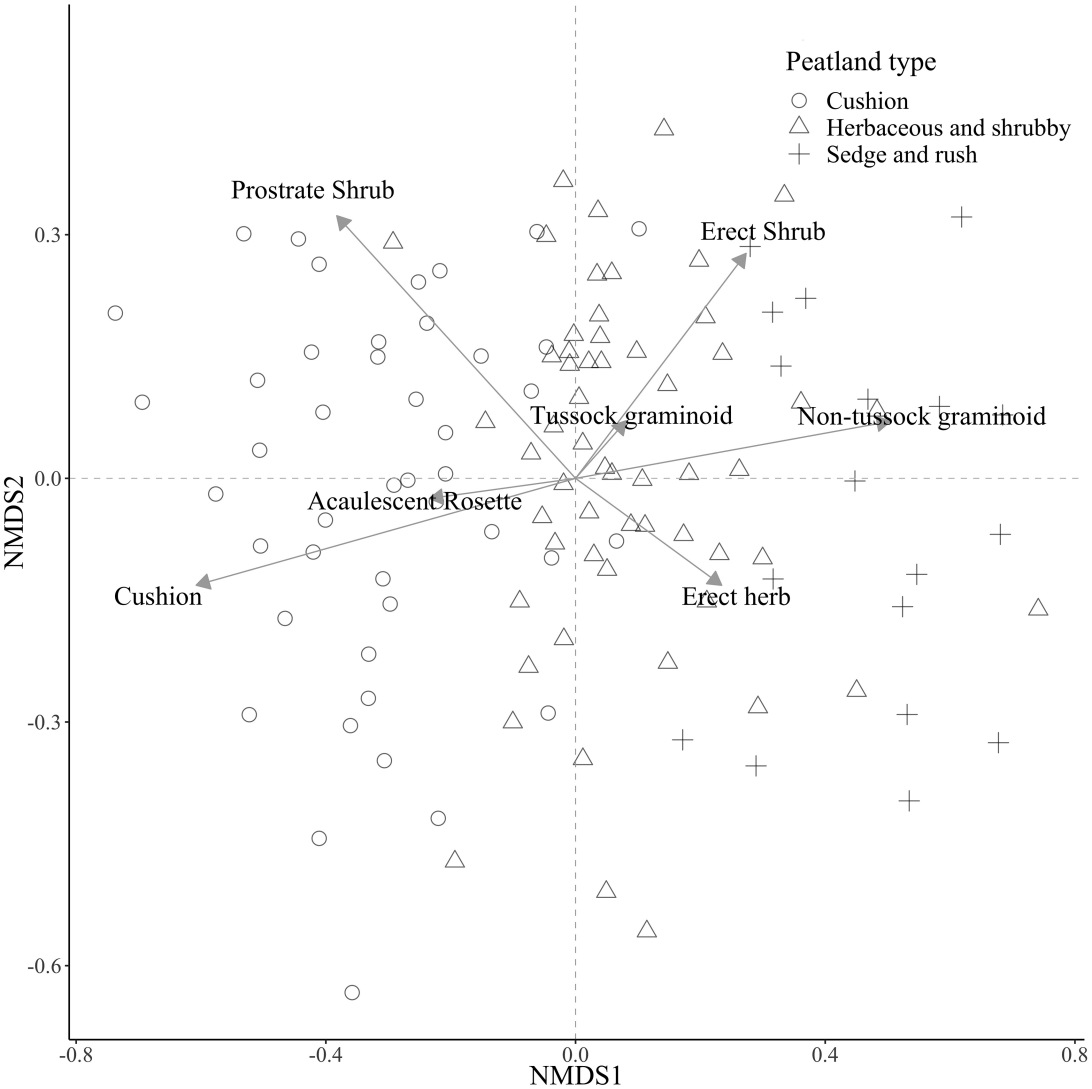
Results of a Non-metric Multidimensional Scaling (nMDS) performed on the vegetation cover of 128 1-m plots, collected at 16 peatlands in the páramos of northern Ecuador. Vegetation data from plots was summarized by growth-form following (Ramsay and Oxley, 1997). For details, please see text.

Three distinct peatland vegetation communities were identified by the cluster analysis (**Table 1**) and by the nMDS (**Figure 1**):

**Table 1:** Peatland growth form groups and accompanying group association (p value), indicator (IndVal component *A*) and dominance values (IndVal component *B*) based on data from 120 peatland plots within Cayambe-Coca National Park and its buffer zone.

			IndVal component A		IndVal component B	
	Growth form	p value	Value	Confidence interval (95%)	Value	Confidence interval (95%)
**[1]** **Cushion peatlands**	Cushion	0.001	0.842	[0.805, 0.877]	1.000	[1.000, 1.000]
	Prostrate shrub	0.005	0.525	[0.397, 0.641]	0.795	[0.682, 0.911]
**[2]** **Herbaceous and shrubby peatlands**	Tussock graminoid*	0.001	0.567	[0.495, 0.654]	0.966	[0.909, 1.000]
	Erect shrub	0.014	0.453	[0.344, 0.565]	0.897	[0.810, 0.967]
	Acaulescent rosette	0.016	0.469	[0.376, 0.580]	0.914	[0.836, 0.982]
	Prostrate herb	0.026	0.419	[0.361, 0.469]	1.000	[1.000, 1.000]
	Trailing herb	0.028	0.541	[0.416, 0.665]	0.621	[0.479, 0.741]
	Herbaceous lycophyta	0.217	0.596	[0.387, 0.725]	0.0.466	[0.333, 0.607]
	Basal rosette	0.503	1.000	[0.000, 1.000]	0.034	[0.000, 0.081]
**[3]** **Sedge and rush peatlands**	Non-tussock graminoid**	0.001	0.700	[0.660, 0.742]	1.000	[1.000, 1.000]
	Erect herb	0.001	0.555	[0.457, 0.655]	1.000	[1.000, 1.000]
	Moss	0.073	0.381	[0.330, 0.418]	1.000	[1.000, 1.000]

*Tussock graminoid includes *Calamagrostis* spp., *Festuca* spp., and *Cortaderia* spp.

**Non-tussock graminoid includes species of Cyperaceae (*Carex* spp., *Eleocharis* spp., and *Uncinia* spp.) and Juncaceae (*Juncus* spp.).

Component *A* is defined as the mean abundance of the given growth form per group divided by the sum of the mean abundances of that same growth form across all groups, while component *B* is defined as the mean relative abundance (De Cáceres and Legendre, 2009).

*Group 1 (Cushion peatlands)*: dominated by cushion (*Plantago rigida* and *Distichia muscoides*) and prostrate shrub growth forms. The cushion growth form is indicative of this group (p= 0.001; IndVal A= 0.842).

*Group 2 (Herbaceous and shrubby peatlands)*: dominated by tussock graminoids (*Calamagrostis* spp. and *Cortaderia* spp.), erect shrubs, prostrate herbs, and acaulescent rosettes, this group is not uniquely indicated by any of the 12 growth forms.

*Group 3 (Sedge and rush peatlands)*: dominated by non-tussock graminoids (*e.g. Carex* sp. and *Juncus* sp.) and moss growth forms, the non-tussock graminoid growth form is indicative of this group (p= 0.001; IndVal A= 0.700).

**Figure 2:**
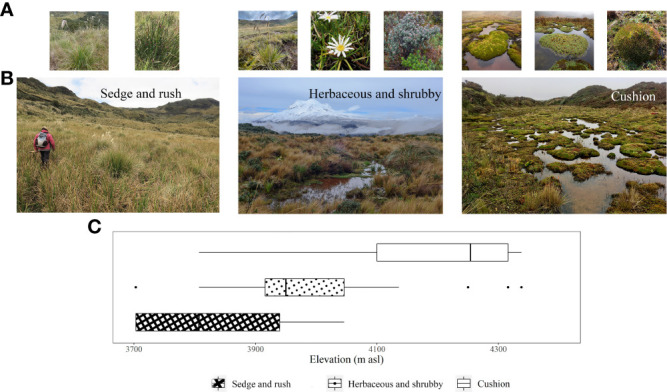
Examples and altitudinal distribution of the three main vegetation groups found in peatlands in the páramos of northern Ecuador. For each vegetation groups, the Figure depicts: **(A)** examples of characteristic species, **(B)** Representative images, and **(C)** Box-plot of the altitudinal distribution.

**Figure 3:**
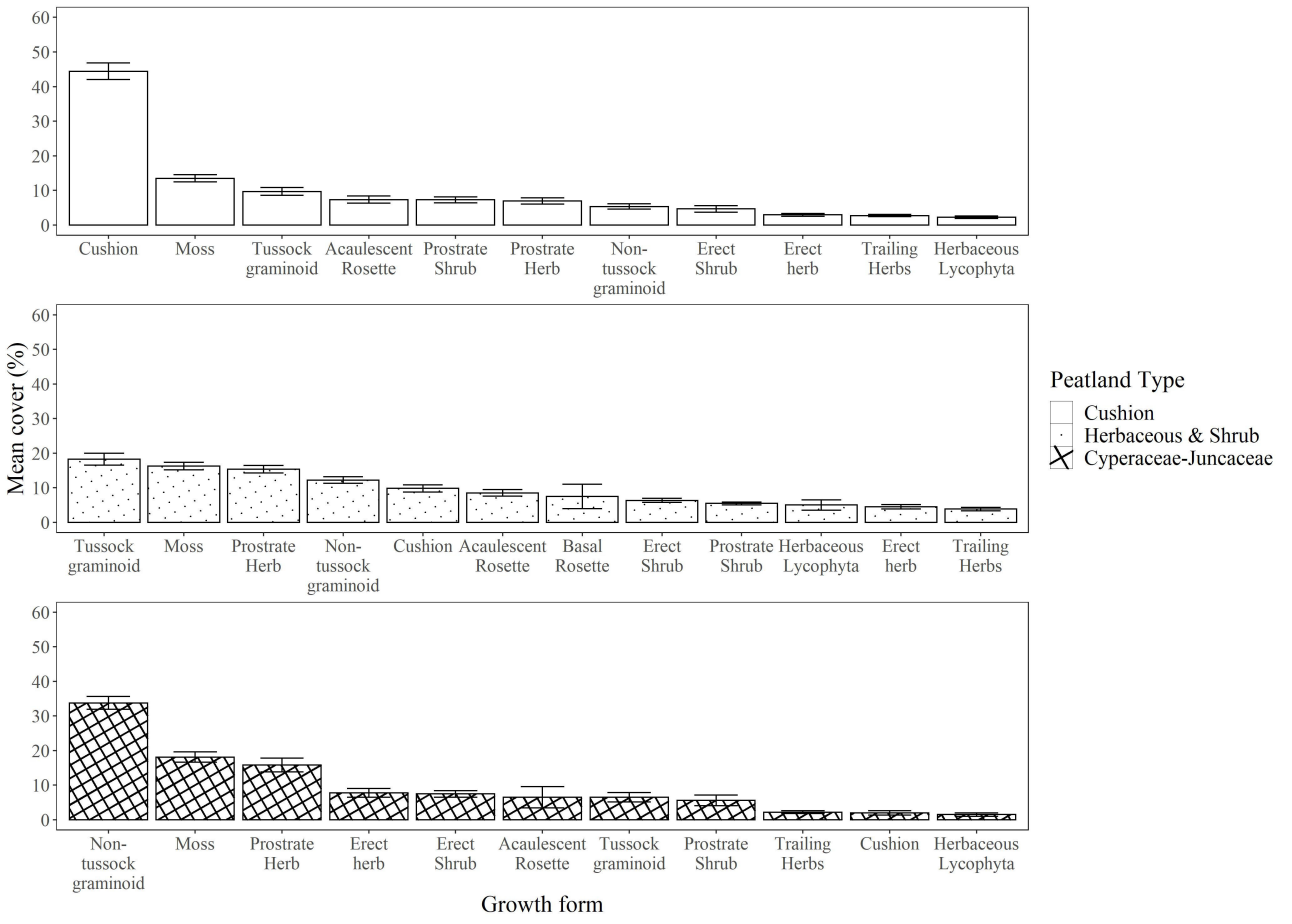
Dominance distribution of the three main vegetation groups found among 16 peatlands in the páramos of northern Ecuador. Dominance in each group was characterized through the mean percent ground coverage of growth-forms (*sensu* Ramsay and Oxley, 1997) derived from 128 1-m plots sampled at 16 individual peatlands.

The cushion peatland type tends to be found at higher elevations (>4100 m, **Figure 2**) and is characterized by high cover of cushion forming species (mean cover >40%, Indval B = 1.000) interspersed with a mix of other growth forms including moss, tussock graminoid, acaulescent rosettes, and prostrate shrubs. Sedge and rush peatlands dominate at lower elevations (<3950 m) and are predominantly covered by non-tussock graminoids (mean cover >30%, Indval B = 1.000) mixed with moss and herbs. Herbaceous and shrubby peatlands occur at intermediate elevations (3950 to 4050 m) and are characterized by heterogeneous plant communities with relatively equal cover of most growth forms. Moss represented the second most dominant growth form in all three peatland types (**Figure 3**).

Despite large differences in plant community structure, mean species richness was similar between the three peatland groups, with 19 ± 2.7 species at Sedge and rush peatlands, and 22.6 ± 2.04 and 23 ± 3.6 species at Cushion and Herbaceous and shrubby peatlands, respectively.

### Aboveground biomass

Of the four peatlands analyzed for aboveground biomass, two fall within the Herbaceous and shrubby peatland category, and two within the cushion peatland category (**Figure 2**). Aboveground biomass decreased with increasing elevation (**Table 2**). Specifically, aboveground biomass at 3920 m (mean ± SE, 27 ± 17.6 Mg ha^-1^) was at least eight times greater than the biomass at higher elevations (1.7 ± 0.7 to 3.3 ± 1.0 Mg ha^-1^), representing an 85% decrease in biomass over 380 m of elevation. The relative contribution of different growth forms to total aboveground biomass also differed between elevations. Tussock graminoids accounted for 70 to 86% of the aboveground biomass in the lower sites (3900 m and 4125 m), 25% at 4240 m, and were not present at 4300 m. This sharp decline is due predominantly to the disappearance of two common tussock-forming species found in the lower sites: *Cortaderia nitida* and, to a lesser extent, *Cortaderia sericantha*. These tussock graminoids are replaced by lower stature vegetation—predominately cushion plants—in higher elevation sites (**Table 2**). Cushion plant contribution to aboveground biomass was negligible at 3920 and 4125 m but became a dominant functional group at the higher sites, contributing between 55 and 60% of the total peatland aboveground biomass.

**Table 2:** Mean growth form percent cover and aboveground biomass contribution in four peatlands located along an altitudinal gradient in the Northeastern Andes of Ecuador.

Elevation	Mean cover (%)	n	Standard Error	Aboveground biomass % contribution
3920 m (27.6 ± 17.6 Mg ha^-1^)				
Erect herbs	8.92	22	3.89	0.2
Basal rosette	4.09	22	3.09	3.7
Low vegetation*	41.43	22	4.33	8.3
Shrubs	2.14	22	0.90	1.3
Tussock graminoids	17.57	22	4.25	18.5
Cortaderia nitida**	25.85	22	4.46	68.1
4125 m (9.7 ± 6.6 Mg ha^-1^)				
Cushion plants	0.90	11	0.62	0.4
Erect herbs	13.00	11	5.86	0.6
Low vegetation	53.34	11	4.89	25.8
Shrubs	1.47	11	0.76	2.0
Tussock graminoids	31.29	11	4.04	71.3
4240 m (1.7 ± 0.7 Mg ha^-1^)				
Cushion plants	59.95	16	10.58	54.8
Low vegetation	23.32	16	6.86	11.3
Shrubs	5.50	16	2.21	7.5
Tussock graminoids	11.23	16	3.00	26.3
4300 m (3.3 ± 1.0 Mg ha^-1^)				
Mixed cushion plants***	4.78	10	3.83	4.2
Low vegetation	65.89	10	9.53	39.7
*Distichia muscoides*	17.97	10	8.97	28.1
*Plantago rigida*	11.36	10	4.67	28.1

*Low vegetation refers to a layer of mosses and matt-forming species and herbs (usually less than 20 cm in height) that forms a continuous layer. Common species include: Racomitrium crispipilum, Campylopus spp., Gentiana sedifolia, Lachemilla orbiculata, and Halenia spp.

**Cortaderia nitida tussock graminoids were considered as a separate category to account for the considerably larger size of this species as compared with other tussock forming species.

***In addition to mono-specific cushion plants of Distichia muscoides and Plantago rigida, at this elevation there were cushion plants in which the two species occurred in complex mixtures that were very difficult to quantify. The percent cover of these cushion plants was quantified separately.

## Discussion

The peatland vegetation types that we found in this study are similar to those identified through a large-scale mapping effort conducted in the same region (Hribljan et al., 2017), with one specific distinction. While Hribljan et al. (2017) used the category “grass peatlands”, here we suggest a more general descriptor (Herbaceous and shrubby) to reflect the more complex and heterogeneous nature of the plant communities within this category. The other two categories (Cushion, Sedge and Rush peatlands) are similar to those described before, and also coincide with vegetation types described for high-elevation peatlands in the Colombian páramo (Benavides et al., 2023).

Vegetation structure across our sites is heterogeneous and seems strongly related to the steep altitudinal gradient of this region. At the lower portion of this gradient (< 3950 m) two types of peatlands were found: “Sedge and rush peatlands”, typically dominated by species of the genera *Carex*, *Eleocharis* and *Juncus*, and “Herbaceous and shrubby peatlands”, with a structurally diverse plant community in which tussock graminoids, mosses, herbs, acaulescent rosettes, basal rosettes, and erect shrubs form a complex matrix without clear dominance of any functional group. As these vegetation types can be found at the same elevations and, sometimes, even within the same site, their different structure probably reflects the influence of other environmental factors such as water table level, or water chemistry. Although in this study we did not measure these factors at all our sites, water table regimes might partially explain these differences, as high and constant water-table levels tend to favor the dominance of species such as *Carex*, *Eleocharis* and *Juncus*, which are known for their capacity to endure prolonged flooding (Ewing, 1996; Hough-Snee et al., 2015). This observation is also supported by a previous study in the same area which reported that a Herbaceous and shrubby peatland, and sedge dominated peatland, separated by less than 200 m, had mean water table levels of 2.8 ± 0.43 cm and -7.6 ± 0.73 cm, respectively, implying that the sedge dominated site was almost permanently flooded and had nearly 10 cm of difference in mean water table level in relation to the Herbaceous and shrubby peatland (Suárez et al., 2022b).

At higher elevations, shrubs and grasses become less frequent and cushion plants -especially *Distichia muscoides* and *Plantago rigida*, dominate the community structure, resulting in a physiognomy which closely resembles that of some of the “*Distichia* peatlands” previously described for the Colombian páramo, and for the jalca and puna ecozones of Central and southern Perú, Bolivia, and northern Argentina (Maldonado-Fonken, 2014; Ruthsatz et al., 2020; Domic et al., 2021; Benavides et al., 2023), whereas, our low elevation sites roughly coincide with the structure of the “peatlands with mosses and shrubs” described from lower jalca sites (3719-3890 m) and from the bofedales of the Central Andes (Navarro and Maldonado, 2002; Maldonado-Fonken, 2014). Similarities in plant community structure are also apparent when comparing our sites with the general descriptions provided by Deil et al. (2011) and Benavides et al. (2023). For example, our higher sites (> 4200 m) exhibit the same matrix species (*D. muscoides*, *P. rigida*) of the hard cushion mires of the Andean belt (Squeo et al., 2006; Deil et al., 2011). These similarities in plant community structure across a gradient that spans at least 11 degrees of latitude suggest that elevation is an important driver of the distribution and functioning of plant communities in high-elevation Andean peatlands across a wide range of environmental conditions. The elevation pattern emphasized here does not exclude possible effects of water chemistry and other environmental factors on plant community structure. For example, semi aquatic environments are often considered as azonal, which means that water saturation patterns might be the driving factors of their structure and functioning (Cooper et al., 2010). From this perspective, further research is needed to explore the relative influence and potential interactions between elevation, water-table patterns, and water chemistry across different scales in these Andean peatlands.

The classification of peatland types suggested by our analysis should not be taken as a definitive and exhaustive description of unique peatland categories in the páramo of Ecuador. In very large peatlands, for example, more than one vegetation type could be present, with Sedge and rush communities dominating areas with higher water table, and Herbaceous and shrubby vegetation in the transition towards the upland páramo. Moreover, our results are strictly limited to the northern Ecuadorian Andes, a region developed on young volcanic soils. Observations in peatlands developed on different geological and climatological settings of the northern and southern Andes suggest that additional types exist including peatlands dominated by thick carpets of *Sphagnum* mosses, and other which are covered by dense stands of shrubs (*Loricaria* spp., *Hypericum* spp., and *Linochilus* spp.). Additional studies are needed across the region to confirm and complement the physiognomic groups that we have described here for the northern Ecuadorian Andes.

As expected for high-elevation peatlands, our results show that our sites are characterized by low carbon storage in the aboveground biomass pool, especially when compared with the large storage capacities (> 2000 Mg ha^-1^) that have been reported for the peatlands in this region (Hribljan et al., 2017). The values that we estimated at our study sites (3 to 27 Mg ha^-1^) are very similar to typical values reported for temperate non-forested (1.36 to 23 Mg ha^-1^) and tropical high-elevation peatlands (8 to 27 Mg ha^-1^) (Dyck and Shay, 1999; Murphy et al., 2009; Murphy and Moore, 2010; Zerbe et al., 2013). Moreover, despite exhibiting very different plant community structure, aboveground biomass values at our peatland sites were also within the range of values (7 to 30 Mg ha^-1^) previously reported for upland páramo ecosystems (Cardozo and Schnetter, 1976; Beekman and Verweij, 1987; Lutz and Vader, 1987; Ramsay and Oxley, 2001). However, the large biomass differences that we found across vegetation types along the altitudinal gradient, raise questions about the role of different plant functional groups or species in terms of forming an protecting the thick layers of peat that characterize these sites.

Together with the large changes in plant community structure and composition across the altitudinal gradient, our results also showed an 8-fold reduction in aboveground biomass in the higher peatlands compared to the lower sites (**Table 2**). This pattern could be explained by at least two non-mutually exclusive alternatives. On the one hand, this drastic decline in plant biomass occurs through an altitudinal gradient of only 640 m, suggesting that aboveground carbon storage in high elevation peatlands could be finely controlled by the changes in functional structure of plant communities, as driven by lower temperatures at higher elevations. This suggests that even slight increases in temperature, far smaller than those forecasted by climate change models for the Tropical Andes (Urrutia and Vuille, 2009), could impose profound changes in the structure and function of high-elevation peatlands in the northern Andes. At the same time, our research indicates that cushion plant cover decreases at lower elevation, underscoring the potentially drastic effect warming temperatures could have on high-elevation cushion peatlands. On the other hand, the decrease of aboveground biomass with increasing elevation could be related to the age of the peatlands that we studied along this gradient. As all our peatland sites formed after the retreat of glaciers roughly 10,000 years ago, sites at lower elevations are probably older than sites at the higher reaches of the mountain. In this scenario, the contrasting life-growth composition and aboveground biomass patterns that we found along the elevation gradient could be rather explained by the influence that time could have on nutrient concentration and other chemical characteristics of soils at higher elevations. In fact, Bosnian et al. (1993) suggested that cushion plants could be favored by low nutrient concentrations and low conductivity that are common in the poorly developed soils at higher elevations. The relative importance of these hypotheses cannot be evaluated with the limited geographical scope of this study, suggesting new avenues for further study of the history and vegetation dynamics of these peatlands.

Our study offers a first attempt at characterizing plant communities in high-elevation peatlands in the northern Ecuadorian Andes, with an emphasis on understanding the effects of elevation on the distribution, structure, and above ground biomass patterns of high-elevation peatland plant communities. Our data suggest that the steep elevational gradients characteristic of Andean environments, are crucial in the local structuring of the physiognomy and composition of peatland vegetation. However, the potential effects of geological setting, time of development, hydrology, micro-topography, and land-use, which are likely to influence vegetation patters in these peatlands, were outside the scope of this paper and warrant future research (Benavides et al., 2013; Urbina and Benavides, 2015). Similarly, additional studies are needed to understand the functional roles of different vegetation types on the development of the large soil carbon reservoirs present in these ecosystems.

## Data availability statement

The original contributions presented in the study are included in the article/supplementary material. Further inquiries can be directed to the corresponding author.

## Author contributions

ES: Project conceptualization, field work, data analysis, and writing of first draft of the manuscript. JH: analysis and writing and manuscript review. SC: field work, soil sample processing, vegetation sampling, and manuscript review. KH, VT, JZ: field work. RJ and LD: analysis, writing and review of the manuscript. All authors contributed to the article and approved the submitted version.
